# microRNA levels in paraffin-embedded indolent B-cell non-Hodgkin lymphoma tissues from patients chronically infected with hepatitis B or C virus

**DOI:** 10.1186/1471-2334-14-S5-S6

**Published:** 2014-09-05

**Authors:** Roberto Bruni, Cinzia Marcantonio, Alessandro Pulsoni, Paola Tataseo, Federico De Angelis, Enea Spada, Fabrizio Marcucci, Sara Panfilio, Paolo Bianco, Mara Riminucci, Umbertina Villano, Maria Elena Tosti, Anna Rita Ciccaglione, Alfonso Mele

**Affiliations:** 1 Department of Infectious, Parasitic and Immunomediated Diseases, Istituto Superiore di Sanità, Rome, Italy; 2 Department of Cellular Biotechnologies and Hematology, Sapienza University, Rome, Italy; 3 ASL Avezzano-Sulmona, Transfusional Medicine and Molecular Biology Laboratory, Sulmona, Italy; 4 Scientific Directorate, Regina Elena Cancer Institute, Rome, Italy; 5 Department of Molecular Medicine, Sapienza University, Rome, Italy; 6 National Centre for Epidemiology, Surveillance and Health Promotion, Istituto Superiore di Sanità, Rome, Italy; 7 Associazione Calabrese di Epatologia (ACE), Pellaro (RC), Italy

**Keywords:** Lymphoma, Hepatitis B Virus, Hepatitis C Virus, microRNA, FFPE

## Abstract

**Background:**

Epidemiological evidence links Hepatitis B Virus (HBV) and Hepatitis C Virus (HCV) to B-cell non-Hodgkin lymphoma (B-NHL). These B-NHLs, particularly those associated with HCV, may represent a distinct sub-group with peculiar molecular features, including peculiar expression of microRNAs (miRs).

The aim of the present study was to search for miRs whose level in indolent B-NHL tissues could be associated with HBV or HCV infection.

**Methods:**

Fourteen formalin fixed paraffin embedded (FFPE) tissues from HBV+, HCV+ and HBV-/HCV- indolent B-NHL patients were analyzed for levels of 34 selected miRs by quantitative Real-Time PCR. Reactive lymph nodes (RLNs) from HBV-/HCV- patients were included as non-tumor control. Statistical analysis of output data included Pearson and Spearman correlation and Mann-Whitney test and were carried out by the STATA software.

**Results:**

MiR-92a was decreased exclusively in HBV-/HCV- B-NHLs, while miR-30b was increased in HBV+ and HCV+ samples, though only the HCV+ achieved full statistical significance. Analysis of a small subset of B-NHLs belonging to the same histological subtype (Nodal Marginal Zone Lymphoma) highlighted three miRs associated with HCV infection (miR-223, miR-29a and miR-29b) and confirmed decreased level of miR-92a in HBV-/HCV- samples also when considering this restricted B-NHL group.

**Conclusions:**

Although caution is needed due to the limited number of analyzed samples, overall the results suggest that differences at the miR expression level exist between indolent B-NHLs developed in patients with or without HBV or HCV infection. The identification of three further miRs associated with HCV by analyzing histologically homogeneous samples suggests that variations of miR levels possibly associated with HBV or HCV may be obscured by the tissue-specific variability of miR level associated with the different histological subtypes of B-NHL. Thus, the identification of further miRs will require, in addition to an increased sample size, the comparison of B-NHL tissues with the same histological classification.

## Background

In addition to liver cancer, increasing evidence has linked Hepatitis B Virus (HBV) and Hepatitis C Virus (HCV) to the development of both indolent and aggressive B-cell non-Hodgkin lymphoma (B-NHL). In fact, most epidemiological studies have identified a positive association between B-NHL and HCV and HBV infection [1-4], and a role for both viruses in the pathogenesis of B-NHL has been suggested [5-8].

Further support for HCV playing a role in development of lymphoma has come from reports on the treatment of HCV infection in B-NHL patients [9-12]. In these studies, HCV-associated B-NHLs have been reported to be highly responsive to antiviral therapy: clearance of HCV resulted in remission of B-NHL, and reappearance of infection was associated with relapse of the disease. Indeed, these observations also suggest that (A) HCV is involved not only in driving development but also in maintaining B-NHL and (B) HCV-related B-NHLs have specific biological properties, and consequently molecular features, characterizing them as a subset distinct from HCV-unrelated B-NHLs, despite histological similarity [9-12]. However, the mechanism(s) underlying the role of HCV in the pathogenesis of B-NHLs, as well as the associated molecular alterations, remain(s) to be clarified.

In regards to HBV, a potential link with the pathogenesis of B-NHL in chronic carriers is provided by its ability to infect peripheral blood mononuclear cells, including B lymphocytes [13]. In a recent study, detection of HBV DNA in B-NHL tissues was more frequent than in T-cell NHL or other lymphatic system disease tissues, suggesting that chronic HBV infection in lymph nodes might be associated with B-NHL [14].

MicroRNAs (miRs) are small, non-coding RNAs emerging as key regulators of several cellular processes, including cell growth, development, differentiation and metabolism. Several hundreds of miRs have been characterized and many of them show tissue-specific expression [15]. Although additional mechanisms have been reported, in vertebrates miRs regulate gene expression mainly at the post-transcriptional level, by inhibiting translation of target messenger RNAs by binding to specific, imperfectly complementary, target sites [16-18]. Each miR may regulate hundreds of potential target mRNAs [19].

Evidence for a role of miRs in the pathogenesis of several diseases, including cancer, as well as in the development of innovative tools for molecular diagnosis and prognosis and in the identification of new targets for therapy has been well documented [18-20].

Studies on Formalin Fixed Paraffin Embedded (FFPE) tissues have shown that, in contrast with protein coding mRNAs that are largely degraded, miRs are substantially unaffected by the standard procedures of FFPE tissue preparation [21-23]. The possibility to reliably quantify miRs in slices of FFPE tissues routinely collected for histopathological analysis makes miRs excellent candidates for biomarker discovery using archived FFPE clinical specimens.

Compelling evidence links miRs to HCV biology. MiR-122a, highly and specifically expressed in liver but not in B lymphocytes, binds to two closely spaced target sites in the 5' noncoding region of the HCV genome, resulting in up-regulation of viral RNA levels [24]. In addition, though not documented in B lymphocytes, the potential for HCV to modulate expression of cellular miRs has been documented in liver cancer cell lines *in vitro *[25,26].

Several studies have searched for miRs involved in development of B-NHL but, to our knowledge, only one study has investigated the possible influence of HCV on miR expression in B-NHL, analyzing Splenic Marginal Zone Lymphoma (SMZL), an indolent B-NHL entity with peculiar clinical and molecular features [27]. No study has so far investigated miR expression in other indolent B-NHLs in patients chronically infected by HBV or HCV.

The aim of the present study was to identify miRs whose level in indolent B-NHLs (nodal marginal zone lymphoma (NMZL), follicular lymphoma (FL), small lymphocitic lymphoma (SLL)) could be related to HBV or HCV infection. B-NHLs from patients positive for HBV or HCV are not as frequent as those from negative ones: in Italy approximately 1 out of 20 cases of all B-NHLs may be attributable to HCV infection [3], and even less if one considers only the indolent fraction; similar considerations also apply to HBV. Thus, retrieval of indolent B-NHL FFPE samples from HCV or HBV positive patients is not an easy task and only major hematological centers managing large patient numbers and routinely performing virological diagnosis may provide such rather rare samples. In the present study, the archive of the Hematology Center of the "Umberto I" University Hospital of Sapienza University of Rome, diagnosing about 50 indolent B-NHL cases per year and routinely performing virological diagnosis, was searched for samples from patients positive for HBV or HCV. Available FFPE tissues, including indolent B-NHLs from 5 HBV positive and 4 HCV positive patients and, as controls, 5 indolent B-NHLs and 9 reactive lymph nodes from HBV/HCV negative patients, were then analyzed. The level of 34 selected miRs was quantified by Real-Time PCR.

## Methods

### Samples

Fourteen indolent B-NHL samples (lymph node biopsies) had been collected from patients for diagnostic purposes before any therapeutic treatment and were classified according to the WHO Classification by expert pathologists. Patient and sample data are reported in Table 1. HBV or HCV infection was diagnosed by testing for serological markers of HBV and HCV by commercial kits. FFPE samples for histopathological analysis were obtained by standard procedures.

**Table 1:** Patient and sample data.

Sample ID	HBV or HCV infection	Subtype of B-NHL	Patient Age	Patient Gender
1	HBV	SLL	66	F
2	HBV	FL	49	M
3	HBV	SLL	65	M
4	HBV	NMZL	73	F
6	HBV	FL	55	F
8	HCV	NMZL	74	M
9	HCV	NMZL	73	F
10	HCV	NMZL	66	F
12	HCV	FL	60	M
14	Negative	FL	72	M
15	Negative	SLL	52	F
16	Negative	NMZL	62	M
17	Negative	NMZL	69	F
18	Negative	NMZL	76	M

HBV: Hepatitis B Virus; HCV: Hepatitis C Virus; B-NHL: B-cell Non Hodgkin Lymphoma; NMZL: Nodal Marginal Zone Lymphoma; FL: Follicular Lymphoma; SLL: Small Lymphocytic Lymphoma; M: Male; F: Female.

The study was approved by the Ethical Committee of the participating Centers. Informed consent was obtained from the patients. The study is in accordance with the ethical standards of the institutional committees and with the Helsinki Declaration.

Nine lymph nodes with minor reactive changes (Reactive Lymph Nodes, RLNs) were also included in the study as non-neoplastic controls. RLNs were analyzed exclusively for the level of the 5 miRs (miR-92a, miR-30b, miR-223, miR-29a and miR-29b) that showed association with presence/absence of HBV or HCV infection in B-NHL patients.

### Total RNA extraction from FFPE tissue sections and quantification

Total RNA was extracted from FFPE tissue samples by the miRNeasy FFPE kit (QIAGEN), according to the manufacturer's instructions. Further details are reported in additional file 1. RNA concentration was determined by the Agilent RNA 6000 Nano Kit on an Agilent 2100 Bioanalyzer (Agilent Technologies), and was calculated as the mean value from two independent runs. Accurate RNA concentration estimate was included to avoid normalization of Real-Time PCR data: normalization was shown to be unnecessary if accurate estimation of RNA concentration is carried out [28].

### miR quantification by Real-Time PCR

The list of assayed miRs is reported in Table 2. A set of 34 miRs was selected for quantification including (A) the 33 most abundantly expressed miRs from the so called "B-cell miRNome", reporting miR expression from three normal B-cell types (naïve, memory and germinal center centroblasts) and from a Burkitt lymphoma cell line [29] and (B) miR-26b, which was included despite the very low level in B-cell miRNome, because of its recently reported association with HCV in SMZL [27]. Further details are reported in additional file 1.

**Table 2:** List of the assayed 34 miRs.

hsa-let-7a	hsa-miR-138	hsa-miR-16	hsa-miR-21	hsa-miR-29b
hsa-let-7b	hsa-miR-140-3p	hsa-miR-17-5p	hsa-miR-221	hsa-miR-29c
hsa-let-7f	hsa-miR-142-3p	hsa-miR-191	hsa-miR-223	hsa-miR-30b
hsa-let-7g	hsa-miR-142-5p	hsa-miR-193b	hsa-miR-25	hsa-miR-30c
hsa-let-7i	hsa-miR-150	hsa-miR-19a	hsa-miR-26a	hsa-miR-339-5p
hsa-miR-106b	hsa-miR-15a	hsa-miR-19b	hsa-miR-26b ^*^	hsa-miR-92a
hsa-miR-125b	hsa-miR-15b	hsa-miR-20a	hsa-miR-29a	

* not selected from B-cell miRNome; it was included because found to be down-regulated in SMZLs from HCV positive patients [27].

miR level was analyzed by Real-Time PCR in 96-well plates with TaqMan commercial assays (Applied Biosystems). In order to exclude artifacts due to inter-run variations, all B-NHL samples were assayed for each miR in the same Real-Time PCR run. Triplicate reactions were carried out for each miR/sample. Output raw Ct data from Real-Time PCR were processed by EXCEL functions, including simple statistical analysis. Processed data were analyzed for statistical significance by the STATA software.

## Results

### Reliability of data from FFPE B-NHL tissues

Total RNA extracted from 14 FFPE B-NHLs was analyzed for 34 selected miRs by commercial Real-Time PCR assays. Patient and sample data are reported in Table 1, and the list of assayed miRs in Table 2.

As a first step, the reliability of the obtained Real-Time PCR data was evaluated. The expression of several miRs is so exquisitely tissue specific that it is being used to trace the tissue of origin of metastases from unknown primary tumors [30-32]. We took advantage of a previous study reporting the miR levels from 40 normal human tissues, including lymph node, in which miRs were quantified by the same Real-Time PCR method used in the present study; the raw Ct values were available as supplementary information [33]. In that study, total RNA samples from commercial sources, extracted from frozen tissues, were used.

The average Pearson correlation between the levels of the 34 miRs in our set of 14 B-NHLs and their levels in 40 human tissues is shown in additional file 2. B-NHLs exhibited high correlation with lymph node (mean r = 0.76 ± 0.09), low/moderate correlation with thymus and spleen (mean r = 0.44 ± 0.11 and 0.42 ± 0.09, respectively) and little if any correlation with the remaining tissues (mean r < 0.3 ± 0.10). This result suggests good reliability of our data from FFPE B-NHL tissues, as they show the expected correlation with data from frozen immune and other tissues reported in a previous independent study.

In addition, a recent study reported a miR signature allowing to distinguish NMZLs from FLs: the levels of miR-221, miR-223 and let-7f, as determined by Real-Time PCR assays, were increased in NMZLs compared with FLs [34]. The median expression of these three miRs was compared in our NMZL cases (n = 7) vs. FL cases (n = 4). Increased miR-221 and miR-223 levels were observed in NMZLs (4.9 and 4.4 fold, respectively), while similar levels of let-7f were present in both subtypes (1.1 fold in NMZLs vs. FLs); however, analysis of let-7f values from individual samples showed an outlier in one of the four FL cases (sample ID: 6): by excluding this sample, 3.2 fold higher level was present in NMZLs *vs*. the remaining FLs (data not shown). It can be concluded that the previously described signature of NMZLs vs. FLs is essentially observed also in the present study.

### miRs differentially expressed in B-NHLs from HBV or HCV infected vs. uninfected patients

The level of the 34 miRs was then analyzed to search for a possible differential expression in association with presence or absence of HBV or HCV infection. B-NHLs from HBV positive patients (HBV+ B-NHLs) (n = 5) and from HCV positive patients (HCV+ B-NHLs) (n = 4) were compared with the control B-NHL group from patients negative for HBV or HCV infection (Ctrl B-NHL) (n = 5).

**Figure 1:**
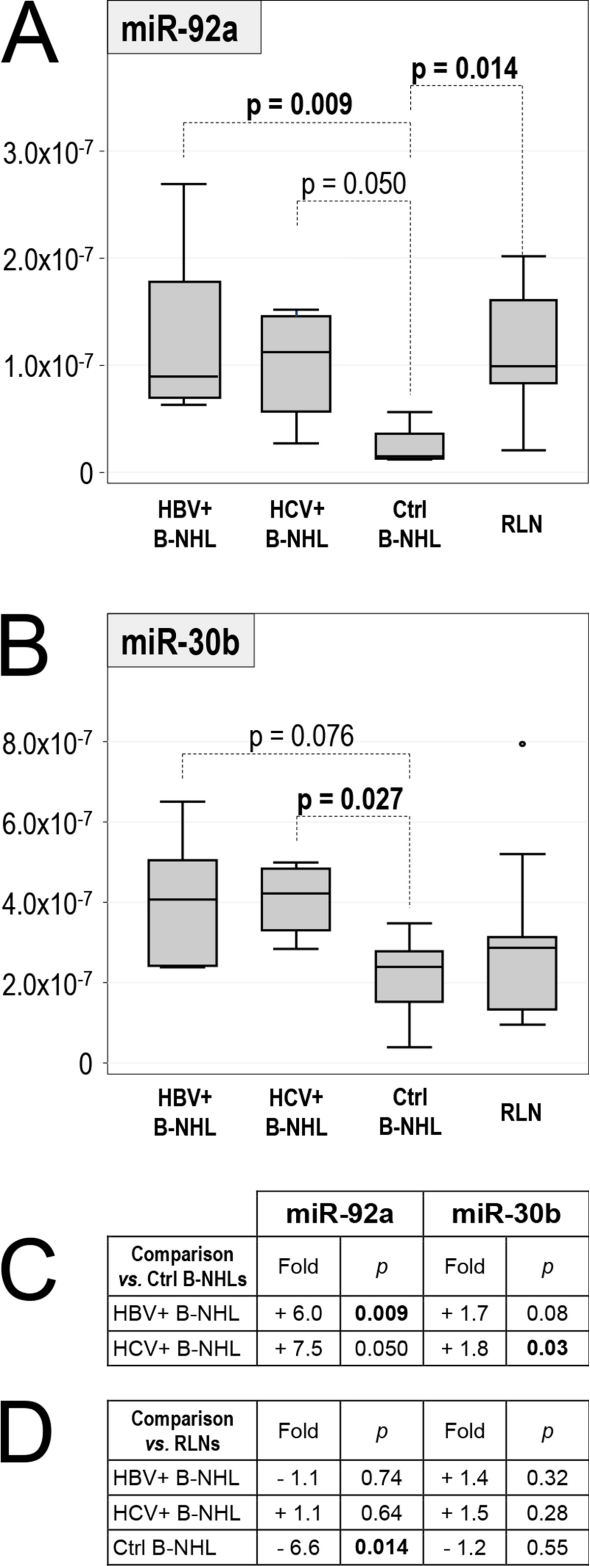
**Level of miR-92a and miR-30b in HBV+ B-NHL, HCV+ B-NHL, Ctrl B-NHL and RLN groups**. Box-plot representation of the level of miR-92a (**A**) and miR-30b (**B**) in HBV+ B-NHL, HCV+ B-NHL, Ctrl B-NHL and RLN sample groups. HBV+ B-NHL: B-NHLs from HBV positive patients; HCV+ B-NHL: B-NHLs from HCV positive patients; Ctrl B-NHL: control B-NHLs from patients negative for both HBV and HCV. The level is reported as 2^-Ct ^values. p values < 0.1 are reported, and those < 0.05 are shown in bold. **C **and **D**: summary of fold-change (*vs*. reference Ctrl B-NHL and RLN, respectively) and p-value (Mann-Withney test). Fold-change was calculated as ratio between median level of each group *vs*. median level of the reference group.

Most miRs, including miR-26b previously reported to be associated with HCV in SMZLs [27], did not show significant differences, except two of them, miR-92a and miR-30b, with p-values achieving or approaching statistical significance (Figure 1A-B; fold vs. control B-NHLs and p values are summarized in Figure 1C). The median levels of miR-92a and miR-30b in HBV+ and HCV+ B-NHLs were higher than in control B-NHLs (miR-92a: 6.0 fold and 7.5 fold, respectively; miR-30b: 1.7 fold and 1.8 fold, respectively).

The level of miR-92a and miR-30b was then analyzed in reactive lymph nodes (RLNs) and compared with the levels in the B-NHL groups (Figure 1 A-B; folds and p values are summarized in Figure 1D). The median level of miR-92a in both HBV+ and HCV+ B-NHLs did not differ from that observed in RLNs (p = n.s.), while in Ctrl B-NHLs it was 6.6 fold lower (p = 0.014) (Figure 1 A, D). In contrast, the median level of miR-30b in HBV+ and HCV+ B-NHLs, though not reaching statistical significance, was 1.4 and 1.5-fold higher than in RLNs, respectively, while in Ctrl B-NHLs it was similar to that observed in RLNs (Figure 1 B, D).

### Correlated expression of miR-30b and miR-92a in B-NHLs

The observed pattern of miR-92a and miR-30b in B-NHLs and RLNs suggested that the levels of these miRs could be correlated and, thus, this possibility was formally evaluated by Spearman (ρ) statistical test.

miR-30b and miR-92a levels were found to be correlated in B-NHLs (ρ = 0.73; p = 0.003), but not in RLNs (ρ = -0.42; p = 0.26). In B-NHL subgroups, statistically significant and very high correlation of miR-30b and miR-92a levels was found in Ctrl B-NHLs (ρ = 0.90; p = 0.037), moderate/high correlation, but not statistically significant, in the HBV+ B-NHLs (ρ = 0.70; p = 0.19) and no significant correlation in the HCV+ B-NHLs (ρ = -0.20; p = 0.80), apparently suggesting that correlation is absent in HCV+ B-NHLs. However, examination of miR-92a and miR-30b levels in individual samples showed that in the HCV+ group the absence of correlation was due to the individual FL sample, while the remaining three NMZLs showed a clearly correlated expression trend (data not shown), suggesting that heterogeneity between subtypes may result in decreased correlation.

To evaluate if histological subtype could be a factor contributing to the correlated expression of miR-92a and miR-30b, correlation was also analyzed in B-NHLs grouped by histological subtype. In SLL, correlation and statistical significance could not be formally evaluated because only three samples were available, not allowing a reliable Spearman test, but examination of miR-92a and miR-30b levels in individual SLL samples showed a correlated expression trend (data not shown). In FL, miR-30b and miR-92a levels were highly correlated, though not reaching statistical significance (ρ = 0.80; p = 0.20), while in NMZL they were very highly correlated and statistically significant (ρ = 0.96; p = 0.0005).

### Search for miRs associated with HCV infection in NMZLs

As suggested by the above analysis, and also from a theoretical point of view, a comparison of histologically homogeneous samples might be more suitable to detect virus-associated hallmarks, because this approach would exclude the variability related to histological differences.

Due to the small number of available samples, this analysis could be carried out only for NMZLs, and in relation with HCV but not HBV: three NMZLs from HCV-positive patients (HCV+ NMZLs) were compared with three control NMZLs from HCV-negative patients (Ctrl NMZLs).

**Figure 2:**
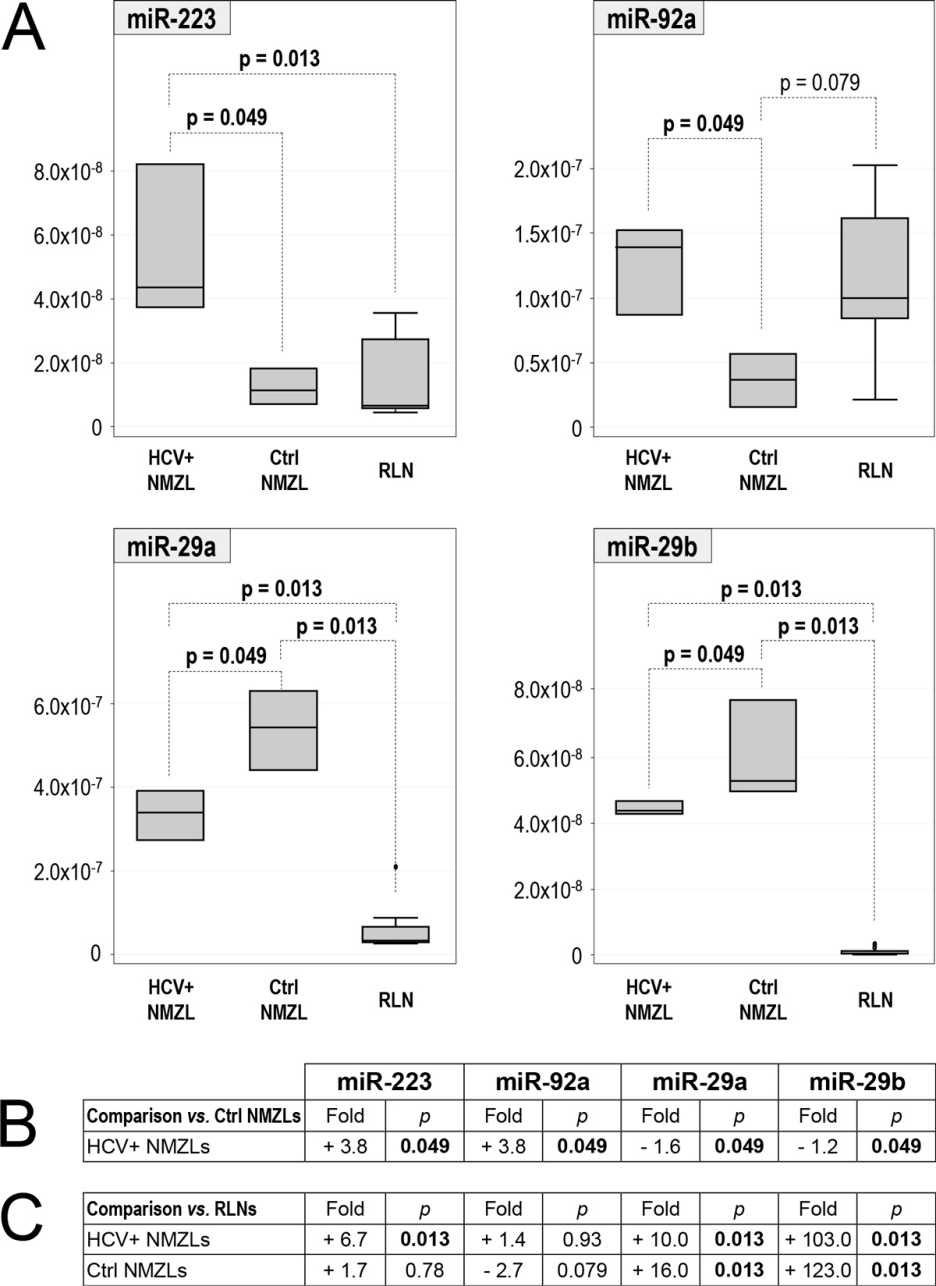
**Level of miR-223, miR-92a, miR-29a and miR-29b in HCV+ NMZL, Ctrl NMZL and RLN groups**. **A**: box-plot representation of the level of miR-223, miR-92a, miR-29a and miR-29b in HCV+ NMZL, Ctrl NMZL and RLN sample groups. HCV+ NMZL: NMZLs from HCV positive patients; Ctrl NMZL: NMZLs from HCV negative patients; RLN: non-neoplastic Reactive Lymph Nodes. The level is reported as 2^-Ct ^values. p values <0.1 are reported, and those < 0.05 are shown in bold. **B **and **C**: summary of fold-change (*vs*. reference Ctrl NMZLs and RLNs, respectively) and p-value (Mann-Whitney test). Fold-change was calculated as ratio between median level of each group *vs*. median level of the reference group.

Four out of the 34 assayed miRs showed a statistically significant different level between the groups (Figure 2): in HCV+ NMZLs, median levels of both miR-223 and miR-92a were 3.8-fold higher than in Ctrl NMZLs (p = 0.049 for both miRs), while miR-29a and miR-29b were 1.6- and 1.2-fold lower, respectively, than in Ctrl NMZLs (p = 0.049 for both) (Figure 2 A-B).

Then, the levels of miR-223, miR-92a, miR-29a and miR-29b were determined in nine RLN controls and compared with those in HCV+ and Ctrl NMZLs.

miR-223 level in HCV+ NMZLs was 6.7-fold higher than in RLNs (p = 0.013), while in Ctrl NMZLs it did not show a significant difference (p = 0.78) (Figure 2 A, C): thus, miR-223 was up-regulated in HCV+ but not in Ctrl NMZLs.

miR-92a level in HCV+ NMZLs showed no significant difference *versus *RLNs (p = 0.93) while in Ctrl NMZLs it showed a 2.7-fold lower level than in RLNs (tending to significance, p = 0.079) (Figure 2), a result similar to that obtained by the above analysis of all available samples, in which B-NHLs negative for HBV and HCV (that included the three NMZLs of the present comparison) showed lower miR-92a levels than RLNs (see previous paragraphs and Figure 1).

Finally, miR-29a and miR-29b levels in both HCV+ and Ctrl NMZLs were significantly higher than in RLNs (p = 0.013 in both comparisons): however, the degree of up-regulation in the HCV+ group was significantly lower than in the HCV- Ctrl group (miR-29a: 10-fold *vs*. 16-fold respectively, p = 0.0495; miR-29b: 103-fold *vs*. 123-fold respectively, p = 0.0495) (Figure 2 A, C).

Then, the comparison *versus *RLNs of miRs up-regulated in HCV+ NMZLs (miR-223, miR-92a, miR-29a and miR-29b) was extended to the whole group of fourteen B-NHLs. In contrast to miR-223 and miR-92a, showing no significant differences, the increased miR-29a and miR-29b level observed in both HCV+ and Ctrl NMZLs was common to all other B-NHLs too: median level in B-NHLs was significantly higher than in RLNs (miR-29a: 11-fold, p = 0.002; miR-29b: 106-fold, p = 0.0002) (data not shown).

In addition, correlation analysis by the Spearman test showed that the miR-29a and miR-29b levels were highly correlated in B-NHLs (ρ = 0.916, p < 0.0001), but not in RLNs (ρ = -0.583, p = 0.099). In B-NHL histological sub-groups, high correlation values were found in the HBV+ and Ctrl B-NHL subgroups (ρ = 0.80 in both groups, albeit not reaching statistical significance: p = 0.10 in both) but not in the HCV+ subgroup (ρ = 0.20, p = 0.80). The lack of statistical significance in individual B-NHL subgroups, but not in the whole group of B-NHLs, is likely an effect of the small sample size of the individual subgroups.

## Discussion

FFPE tissues have been reported in previous studies to be as suitable as frozen tissues for quantification of miR expression: this finding was confirmed in the present study. The results of the correlation analysis of Real-Time PCR data from our set of FFPE B-NHLs and data previously reported from 40 different frozen tissues showed the expected correlations: miR profiles from B-NHLs were highly correlated with profiles from lymph node tissue, to a lesser extent with spleen and thymus and not at all with the remaining 37 tissues. In addition, a previously described miR signature distinguishing NMZLs from FLs was observed also in samples of the present study.

Association of miR-26b with HCV in SMZLs was reported in a previous study [27]. No significant association of miR-26b with HCV was observed in our set of B-NHLs, that included NMZLs, FLs, SLLs, but not SMZLs. In another lymphoma subtype, Diffuse Large B Cell Lymphoma (DLBCL), it has been reported that its location is an important factor in determining the differential expression of miRNAs: in fact, two miRs (miR-17-5p and miR-127) showed significant level differences depending on the site of presentation (central nervous system, testicular, nodal) [35]. Thus, it is possible that HCV may affect miR-26b in SMZL due to the splenic localization of that B-NHL.

Compared with Ctrl B-NHLs, the level of miR-92a and miR-30b appeared to be increased in HBV+ and HCV+ B-NHLs. However, compared with non-neoplastic RLNs the miR-92a levels appeared unchanged in the HBV+ and HCV+ groups and decreased in B-NHLs negative for HBV and HCV, suggesting down-regulation in Ctrl B-NHLs, rather than up-regulation in HBV+ and HCV+ B-NHLs (Figure 1A). In contrast, the level of miR-30b in HBV+ and HCV+ B-NHLs was increased also with respect to RLNs; thus, miR-30b up-regulation appears to be associated with HBV and HCV, though the relevance of this result is limited by the lack of significance in the comparison with RLNs, a result influenced by the small level of variation and by the limited number of available samples.

Although miR-92a is usually up-regulated in tumors compared to normal tissues [36,37], our finding is not surprising because miR-92a down-regulation in tumors has also been described, e.g. in breast cancer in association with aggressive features and increased tumor macrophage infiltration [38]. This underscores the importance of carefully studying miR expression in specific tumor types, because a miR can show different expression levels in different tumor types.

The unchanged level of miR-92a in HBV+ and HCV+ B-NHLs, as opposed to the decrease in HBV-/HCV- B-NHLs, suggests either that HBV and HCV infection might play an opposite, compensatory role on miR-92a level or that in these patients B-NHL develops in the absence of miR-92a down-regulation.

Levels of miR-92a and miR30b were found to be highly correlated in B-NHLs but not in RLNs, suggesting that the correlation between these miRs is a tumor-associated alteration. This finding implies that the observed levels of miR-92a in HBV+ B-NHLs, HCV+ B-NHLs and RLNs, though similar, actually result from different underlying mechanisms. The correlation of miR-92a and miR-30b was found to be very high and significant in Ctrl B-NHLs, but was less pronounced in HBV+ and HCV+ B-NHLs, suggesting these viral infections may play some role in modifying the correlated expression of these miRs.

Overall, even considering the limited number of available samples, the results appear to suggest that some differences at the miR level indeed exist between indolent B-NHLs from patients with or without HBV or HCV infection. However, they were difficult to detect, mainly because of the small sample size, that negatively affected the statistical significance, but likely also because of the association of miR expression with tissue specificity and differentiation [39]. Indeed, the different miR levels associated with different histological subtypes likely represent a bias able to obscure small changes associated with HBV or HCV infection, preventing the identification of those miRs actually influenced by these viral infections, but whose variation is smaller than that observed among different histological subtypes.

Consistent with this view, analysis of B-NHL tissues belonging to the same histological subtype (NMZL) led to identification of four miRs (miR-92a, miR-223, miR-29a and miR-29b) that showed significantly different levels between HCV+ and HCV- cases, despite the small sample size. Actually, the comparison with RLNs suggested down-regulation of miR-92a in Ctrl NMZLs, rather than up-regulation in HCV+ NMZLs, confirming also in the restricted NMZL subgroup what observed analyzing all B-NHL cases (see above).

In contrast to miR-92a, miR-223 was up-regulated in HCV+ NMZLs: the level was 3.8-folds higher than in Ctrl NMZLs and 6.7-folds higher than in RLNs, suggesting a role for HCV in increasing the miR-223 level.

MiR-29a and miR-29b were up-regulated in both HCV+ and Ctrl NMZLs compared to RLNs, and extension of the analysis to all available B-NHLs showed that up-regulation of these miRs was common to the whole B-NHL group, a feature also shared with another lymphoid malignancy, chronic lymphocytic leukemia [40]. However, up-regulation in the HCV+ NMZLs was significantly lower than in the Ctrl NMZLs, suggesting a role for HCV in "dampening" the up-regulation. This hypothesis is supported by the observation that miR-29 was down-regulated by HCV infection in hepatocytes [41], and a similar effect might also occur in HCV-infected cells other than hepatocytes, such as B-cells.

Intriguingly, both miR-223 and miR-29a, associated with HCV in the present study, have been reported to be activated by a common transcription factor, CEBPA [42-45]. The possible interaction of HCV with this factor, if any, is expected to be complex, because CEBPA leads to increased levels of both miR-223 and miR-29a, while HCV was associated with increased miR-223 but decreased miR-29a.

## Conclusions

Although the significance is hampered by the limited number of available samples, the present study suggests the existence of differences at the miR expression level between indolent B-NHLs developed in patients with or without HBV or HCV infection.

Analysis of histologically heterogeneous B-NHLs showed that miR-30b was increased in HBV+ and HCV+ cases, while miR-92a was decreased in cases negative for these infections; the levels of these miRs proved to be correlated in B-NHLs but not in RLNs.

Analysis of a small subset of B-NHLs with the same histological subtype classification (NMZL) showed association of four miRs (miR-92a, miR223, miR-29a and miR-29b) with HCV infection. Though caution is needed due to the limited number of analyzed samples, nevertheless this result suggests that heterogeneity of histological subtypes may represent an obstacle for the identification of miRs possibly influenced by HBV or HCV. Thus, further studies aiming at identifying miRs associated with HBV and HCV in B-NHLs will require both a larger sample size and the comparison of B-NHL tissues with the same histological subtype classification.

## List of abbreviations

HBV: Hepatitis B Virus; HCV: Hepatitis C Virus; HBV+: HBV positive; HCV+: HCV positive; NHL: Non-Hodgkin Lymphoma; B-NHL: B-cell Non-Hodgkin Lymphoma; NMZL: Nodal Marginal Zone Lymphoma; SMZL: Splenic Marginal Zone Lymphoma; FL: Follicular Lymphoma; SLL: Small Lymphocytic Lymphoma; miR: microRNA; FFPE: Formalin Fixed Paraffin Embedded.

## Competing interests

The authors declare that they have no competing interests.

## Authors' contributions

RB contributed to design research, carried out tissue RNA extraction and quantification, analyzed data, and wrote the manuscript; CM, PT and UV performed miR quantification experiments; AP and FDA collected clinical data and contributed to design research; SP contributed to collect clinical data; MR and PB carried out histopathological analysis and subtype classification of tissue specimens; MET performed statistical analysis; ARC contributed to design research and analyze the data; ES, FM and AM contributed to design research.

## Supplementary Material

Additional file 1 Additional details of methods Click here for file

Additional file 2 Correlation of miR profiles from B-NHLs with miR profiles from 40 different tissues Click here for file

## Declarations

The publication of this supplement was partly supported by an unrestricted grant provided by Roche. The articles were independently prepared by the authors with no input from Roche. Roche was not involved in selecting the articles for the supplement.

This article has been published as part of *BMC Infectious Diseases *Volume 14 Supplement 5, 2014: Proceedings of the Third Workshop of the HCV Study Group in the Calabria Region: Results from the South Italian Network for Rational Guidelines and International Epidemiology (SINERGIE) Project. The full contents of the supplement are available online at http://www.biomedcentral.com/bmcinfectdis/supplements/14/S5.
