# Supplementary material for: microRNA levels in paraffin-embedded indolent B-cell non-Hodgkin lymphoma tissues from patients chronically infected with hepatitis B or C virus
Source: BMC Infect Dis. 2014 Sep 5;14(Suppl 5):S6. doi: 10.1186/1471-2334-14-S5-S6 (PMC4160900; doi:10.1186/1471-2334-14-S5-S6)
Supplement: Additional file 1 — Additional details of methods [file 1471-2334-14-S5-S6-S1.pdf]

Additional details of methods

Total RNA extraction from FFPE tissue sections and quantification.

Total RNA was extracted from FFPE tissue samples by the miRNeasy FFPE kit (QIAGEN). In brief, 4 freshly cut microtome slices (15 µm thickness) were subjected to paraffin removal by xylene treatment followed by ethanol washing to remove xylene; RNA was then extracted according to the remaining manifacturer's instructions of the kit.

Eluted RNA concentration was carefully determined by the Agilent RNA 6000 Nano Kit according to the manufacturer instructions on an Agilent 2100 Bioanalyzer (Agilent Technologies). The RNA concentration of each sample was calculated as the mean value from two independent runs on 2100 Bioanalyzer. Accurate RNA concentration estimate was included to avoid normalization of Real-Time PCR data: normalization has been shown to be unnecessary if accurate estimation of RNA concentration is carried out (Hugget et al., 2005).

miR quantification by Real-Time PCR.

Overall, a set of 34 miRs was selected for quantification in B-NHLs. We chose to include the 33 most abundantly expressed miRs from the so called "B-cell miRNome", reporting miR expression from three normal B-cell types (naïve, memory and Germinal Center centroblasts) and from Ramos cells, a Burkitt lymphoma cell line (Basso et al., 2009). This approach was chosen because: (a) highly expressed miRs are more accurately quantified than poorly expressed miRs; (b) due to miR regulator function on target mRNAs, biological effects due to level variations are thought to be stronger for miRs whose copy number per cell is high than for miRs whose copy number per cell is low or barely detectable and (c) the number of miRs to be tested is small enough to be conveniently managed by single Real-Time PCR assays. Based on miR levels reported as "corrected count values" in supplementary information in that study, the 33 miRs showing the highest expression level in at least one of the four analysed cell types were selected (Table 2). Despite being highly expressed, miR-1280 was excluded because it has been demonstrated to be a fragment of a tRNA, not a miR, and removed from miRBase public miR repository. In addition, despite the very low level in B-cell miRNome, miR-26b was also included because of its recently reported association with HCV in SMZL (Peveling-Oberhag et al., 2012).5 The final 34 miR set includes miRs reported to be deregulated in lymphoid malignancies, such as miR-15a, miR-16, miR-21, miR-223, miR-29a, miR-29b and five members of the miR-17-92 cluster (reviewed in Fabbri and Croce, 2011).

miR level was analysed by Real-Time PCR in 96-well plates with TaqMan commercial assays (Applied Biosystems). In order to exclude artifacts due to inter-run variations, all B-NHL samples were assayed for each miR in the same Real-Time PCR run. Triplicate reactions were carried out for each miR/sample. Output raw Ct data from Real-Time PCR were analysed by standard procedures; simple statistical analysis of output data was carried out by EXCEL functions (mean value from triplicate reactions, standard deviation, 2^{-Ct} linear transformation of Ct values, Pearson correlation).

Statistical significance of miRNA level differences was evaluated by Mann-Whitney test (STATA software) on 2^{-Ct} values, i.e. linear transformation of Ct values, according to previous recommendations (Schmittgen and Livak, 2008). P values < 0.05 were considered to be significant.

References

Basso K, Sumazin P, Morozov P, Schneider C, Maute RL, Kitagawa Y et al.. Identification of the Human Mature B Cell miRNome. Immunity 2009; 30(5):744-52.

Fabbri M, Croce CM. Role of microRNAs in lymphoid biology and disease. Curr Opin Hematol. 2011; 18(4): 266-272. doi: 10.1097/MOH.0b013e3283476012.

Huggett J, Dheda K, Bustin S and Zumla A. Real-time RT-PCR normalisation; strategies and considerations. Genes and Immunity 2005; 6, 279–284.

Peveling-Oberhag J, Crisman G, Schmidt A, Döring C, Lucioni M, Arcaini L et al. Dysregulation of global microRNA expression in splenic marginal zone lymphoma and influence of chronic hepatitis C virus infection. Leukemia 2012; 26 (7): 1654-1662. doi: 10.1038/leu.2012.29.

Schmittgen TD, Livak K. Analyzing real-time PCR data by the comparative C(T) method. Nat Protoc. 2008; 3(6):1101-1108.
