# Supplementary material for: microRNA levels in paraffin-embedded indolent B-cell non-Hodgkin lymphoma tissues from patients chronically infected with hepatitis B or C virus
Source: BMC Infect Dis. 2014 Sep 5;14(Suppl 5):S6. doi: 10.1186/1471-2334-14-S5-S6 (PMC4160900; doi:10.1186/1471-2334-14-S5-S6)
Supplement: Additional file 2 — Correlation of miR profiles from B-NHLs with miR profiles from 40 different tissues [file 1471-2334-14-S5-S6-S2.pdf]

Correlation of miR profiles from B-NHLs with miR profiles from 40 different tissues

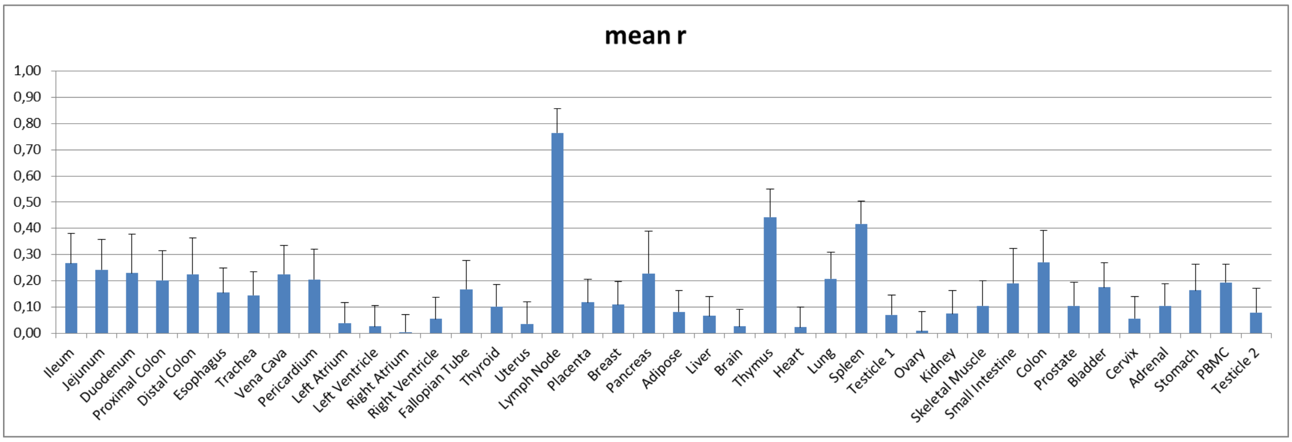

Average Pearson correlation values and standard deviation obtained by comparing the levels of 34 miRs from the 14 B-NHLs of the present study with the corresponding levels in 40 human tissues, reported in a previous study (Liang et al., 2007).
Statistical test was carried out on 2^{-Ct} transformed Ct values, as recommended (Schmittgen and Livak, 2008).

References

Liang Y, Ridzon D, Wong L and Chen C. Characterization of microRNA expression profiles in normal human tissues. BMC Genomics 2007, 8: 166.

Schmittgen TD, Livak K. Analyzing real-time PCR data by the comparative C(T) method. Nat Protoc. 2008; 3(6):1101-1108.
